# Reduction of chickens use to perform *in vitro* pre-screening of novel anticoccidials by miniaturisation and increased throughput of the current *Eimeria tenella* compound-screening model

**DOI:** 10.12688/f1000research.123102.1

**Published:** 2022-10-04

**Authors:** Sara Arias-Maroto, Kelsilandia Aguiar-Martins, Javier Regidor-Cerrillo, Luis Ortega-Mora, Virginia Marugan-Hernandez

**Affiliations:** 1 Department of Pathobiology and Population Sciences, Royal Veterinary College, London, Hatfield, Hertfordshire, AL8 7TA, UK; 2 SALUVET-Innova S.L, Madrid, Madrid, 28040, Spain

**Keywords:** in vitro model, miniaturisation, animal reduction, high throughput, anticoccidial compounds, chicken coccidiosis, Eimeria species

## Abstract

We have developed an *in vitro* model for the evaluation of potential anticoccidial properties of novel compounds aimed to control chicken coccidiosis, a costly disease for the poultry industry. This disease is caused by protozoan parasites of the genus *Eimeria* (Apicomplexa), and it is mainly controlled by chemoprophylaxis with ionophores and chemical anticoccidials; however, there is an overall agreement about the limitation of these traditional drugs and the need to improve current methods of control. Anticoccidial activities of novel compounds is currently evaluated by expensive experiments that involve large numbers of chickens. The use of our *in vitro* model for the pre-screening of essential oils led to a reduction of 67% of the chickens used in the *in vivo* trials for validation. *Eimeria* parasites can only complete their life cycle in their animal host, therefore chickens are required for their propagation as they cannot be propagated *in vitro.* In this study, we describe how further optimisation of this *in vitro* model by miniaturisation can have an additional impact in reduction of the number of chickens used for the generation of parasite stocks for provision of the *in vitro* model. We have estimated that the use of one chicken could support the evaluation of 10 compounds with a 96-well plate format versus only two compounds with a 24-well plate format, which means an 80% reduction in chicken use. In this study we have proved that the miniaturisation into a 96-well plate format perfectly mimics the invasion and replication observed before in the 24-well plate format. In addition, the 96-well plate format has allowed the simultaneous pre-screening of higher numbers of anticoccidial drugs at different concentrations following streamlined protocols in a more cost-effective way, factors that are beneficial for a wider uptake of the model by other researchers investigating anticoccidial compounds.

Research highlights **Scientific benefit:**

• Increased throughput for evaluation of new compounds aimed to control chicken coccidiosis by miniaturising the previously developed *in vitro* compound-screening model

**3Rs benefit:**

• Evaluation of novel anticoccidial compounds will require fewer chickens for the generation of parasite stocks

**Practical benefits:**

• Reduced variability: simultaneous evaluation of a larger number of anticoccidial compounds
• Cost effectiveness: less materials and reagents are necessary per evaluated compounds
• Streamlined protocols: 96-well plate format is used through the whole process (cell culture, DNA isolation and qPCR analysis)

**Current application:**

• High-throughput screening of potential anticoccidial compounds under investigation by commercial companies and/or public research organisations

**Potential applications:**

• Adaptation of equivalent *in vitro* models for other *Eimeria* species causing disease in poultry or livestock

## Introduction

The use of *in vitro* models in biosciences and biomedical research has supported many scientific advances and has emerged as a suitable alternative to reduce time and cost associated with *in vivo* models by replacing and/or reducing the use of experimental animals in many areas. The implementation of *in vitro* models for anticoccidial drugs pre-screening as an alternative to *in vivo* tests is leading to the replacement and reduction of an important number of chickens used in research in coccidiosis control ( Thabet *et al.*, 2017). Chicken coccidiosis is an enteric disease caused by different species of the genus *Eimeria* (Apicomplexa) ( Burrell *et al.*, 2020), characterized by malabsorption, diarrhoea and haemorrhage with an important impact on chicken meat and egg production worldwide that cause >£10 billion per annum losses ( Blake *et al.*, 2020). Coccidiosis has also impacted on the ‘Five Freedoms’ of animal welfare, more in particular affecting the ‘freedom from discomfort’ and ‘freedom from pain, injury’ ( Webster, 2001). Resistance against current anticoccidial drugs, as well as near-prohibition of ionophores antibiotics in some parts of the world and public concerns about the use of drugs in animals for human consumption, has led to an increased research in new substitute compounds, evidenced by the high number of new publications in the area in recent years (>25 *in vivo* studies within the past two years involving >7,000 chickens).

We previously developed an *in vitro* model to evaluate anticoccidial compounds effects in *Eimeria tenella* ( Marugan-Hernandez *et al.*, 2020) and a first direct application for the evaluation of two essential oils ( Sidiropoulou *et al.*, 2020) led to a reduction of the number of chickens used for *in vivo* testing. Results obtained *in vitro* showed that each of the essential oils exhibited anti-parasitic effects at different concentrations. This supported the selection of a single experimental group (90 animals + control group) of a combined treatment at a single dose, excluding the evaluation of the compounds separately since the *in vitro* model had already proved their dose-independent individual effects; therefore, it was not considered necessary to re-evaluated this *in vivo.* The *in vivo* experiment was reduced from six to two experimental groups (test and control), resulting on a reduction of 90×4=360 chickens in a single study (67%). This *in vitro* model has been standardised, applied and published using a 24-well plate format ( Marugan-Hernandez *et al.*, 2020); in this study, we aimed to adapt the model to a 96-well plate format. This miniaturisation will reduce the numbers of parasites needed for compound testing, and therefore the number of chickens used to generate parasites stocks, additionally allowing the evaluation of a higher number of compounds simultaneously. *Eimeria* spp. cannot complete their life cycle *in vitro*; therefore, the natural host (chicken) is necessary to produce stocks for further experimental procedures. The use of one chicken can generate enough initial parasite material (oocysts) for the pre-screening of two compounds in the 24-well plate format. The use of the 96-well plate format will allow the pre-screening of 10 compounds per animal, this has the potential to reduce local animal use for the study of anticoccidial compounds by 80%.

## Methods

### Ethical statement

This study was carried out in strict accordance with the Animals (Scientific Procedures) Act 1986 (United Kingdom Parliament Act). All animal studies and protocols were approved by the Royal Veterinary College Animal Welfare and Ethical Review Board (AWERB) and performed under the United Kingdom Government Home Office under specific project licence (PDAAD5C9D).

### Parasites and chickens

*Eimeria tenella* Wisconsin (Wis) strain ( McDougald & Jeffers, 1976) was propagated chickens. Table 1 summarises the detailed information of the experiment and used animals following ARRIVE guidelines.

**Table 1.** Experimental condition of chickens (ARRIVE guidelines).

Study design	• Single group of chickens for standard amplification of *E. tenella* Wis parasites
Sample size	• Ten chickens were caged in two groups of 5 animals • Stocking density of 5 kg/m ^2^ (Defra maximum legal stocking density is 39 kg/m ^2^)
Inclusion/exclusion criteria	• All animals included
Randomisation	• All animals were used, randomisation does not apply
Blinding	• All animals were used, blinding does not apply
Outcome measures	• Optimal output of *E. tenella* Wis oocysts (~40 million/chicken) at the given dose (4,000/chicken)
Statistical methods	• n/a
Experimental animals	• Breed: White Leghorn – specific pathogen free (from APHA) • Sex: mixed sex • Age: 3-week-old (arrival)/5-week-old (end)
Experimental procedures	• Placed in cages at arrival (no further change will be done) • Acclimation for 1 week • Oral gavage with 4,000 oocysts suspended in 1 ml of distilled water at 4-week-old • Expected severity: Mild • Schedule 1 at 5-weeks-old
Results	• Sufficient oocysts were obtained for provision of the *in vitro* experiments (~400 million)

Pastor-Fernandez *et al.* (2019) provides detailed protocols for oocysts isolation and excystation as well as for sporozoite purification. Sporulated oocysts were stored in water at 4 ^o^C for up to six months, the moment from which they start to lose viability for *in vitro* tests. Freshly purified sporozoites were used to infect cell monolayers immediately.

### Cell culture

Standard protocols for cell culture maintenance were the same described for the standardisation of the *in vitro* model for compound-screening ( Marugan-Hernandez *et al.*, 2020). Briefly, Madin-Darby bovine kidney (MDBK) cells (NBL-1; ECACC-Sigma-Aldrich) were used as host cell cultures and maintained in a 37 ^o^C humidified incubator with 5% CO _2_ atmosphere. Cells were cultured in Advanced Dulbecco’s modified Eagle’s Medium (AdDMEM; Gibco) supplemented with 2% heat-inactivated foetal bovine serum (FBS; Sigma) and 100 U/mL penicillin/streptomycin (Fisher, Leicestershire, UK). Confluent cell monolayers (100%) were passaged twice a week to a new confluence of 70-80% by washing them in Ca ^2+^- and Mg ^2+^-free PBS and released with 3mL 0.25% Trypsin/EDTA (Gibco), incubated for three minutes, followed by neutralisation with 5 mL volume of complete medium. The cell suspension was centrifuged at 1,500 g for 10 minutes at room temperature after which the pellet was suspended in 5 mL of fresh medium. Cells were counted and seeded on T75 flasks (Thermo Fisher Scientific) at a density of 10×10 ^6^ cells with AdDMEM at 15 ml of final volume or seeded on flat bottom 96-well microtiter plates (Thermo Fisher Scientific Nunc) at a cell density of 0.05×10 ^6^ cells in each well in 100 μl of AdDMEM culture media for subsequent infection with sporozoites (see *Optimisation of infection ratios* section).

*Optimisation of infection ratios*

Cells were seeded on flat bottom 96-well microtiter plates (Thermo Fisher Scientific Nunc) at a cell density of 0.1×10 ^6^ or 0.05×10 ^6^ cells in each well in 100 μl of AdDMEM culture media (lower density than 0.3×106 per well in 500 μl used in 24-well plates) for subsequent infection after two hours with sporozoites at different sporozoites:cells ratios (1:1, 2:1, 4:1; Table 2). Three wells were used per conditions (A, B, C) and incubated at 41 ^o^C, 5% CO _2_. After 4 hours, infected monolayers were removed with 3 mL 0.25% Trypsin/EDTA (Gibco) and cells counted in a Fuchs-Rosenthal counting chamber. Empty cells were recorded as non-infected, cells with the presence of at least one sporozoites were recorded as infected. Two counts were done per well ( Table 2).

**Table 2.** Optimisation of conditions for achieving high rates of sporozoite infection of MDBK cells.

Ratio sporozoites:cells (no. sporozoites)	Wells	Non-infected cells	Infected cells	% Infected cells/count	% Infected cells/well	% Infected cells (average)	Standard deviation
**0.05×10 ^6^ cells/well**							
**1:1 (0.05×10** ^**6**^ **)**	A	21	16	43.2	36.1	39.0	7.6
		27	11	28.9			
	B	26	18	40.9	47.6		
		21	25	54.3			
	C	26	13	33.3	33.3		
		22	11	33.3			
**2:1 (0.1×10** ^**6**^ **)**	A	12	23	65.7	60.2	60.7	7.2
		19	23	54.8			
	B	7	21	75.0	68.1		
		12	19	61.3			
	C	22	21	48.8	53.8		
		14	20	58.8			
**4:1 (0.2×10** ^**6**^ **)**	A	7	14	66.7	68.1	73.2	5.2
		7	16	69.6			
	B	10	26	72.2	73.0		
		16	45	73.8			
	C	11	35	76.1	78.5		
		9	38	80.9			
**0.1×10 ^6^ cells/well**							
**1:1 (0.1×10** ^**6**^ **)**	A	22	30	57.7	59.5	57.3	2.0
		22	35	61.4			
	B	21	29	58.0	56.6		
		22	27	55.1			
	C	26	34	56.7	55.7		
		34	41	54.7			
**2:1 (0.2×10** ^**6**^ **)**	A	17	43	71.7	63.5	61.1	7.3
		17	21	55.3			
	B	21	51	70.8	66.9		
		17	29	63.0			
	C	22	34	60.7	52.9		
		33	27	45.0			
**4:1 (0.4×10** ^**6**^ **)**	A	15	33	68.8	75.8	80.0 ^a)^	4.3
		10	48	82.8			
	B	6	28	82.4	79.9		
		7	24	77.4			
	C	8	28	77.8	84.3		
		3	30	90.9			

^a)^ Cells were infected with multiple sporozoites which will compromise cell monolayer stability.

### Anticoccidial drugs

Analytical standard preparations of the classical anticoccidial drugs amprolium hydrochloride (AMP), robenidine hydrochloride (ROB) and salinomycin monosodium hydrate (SAL) were purchased from Sigma (Sigma-Aldrich). Drugs were prepared at 10 mg/mL stock in dimethyl sulfoxide (DMSO; Merck) and dilutions for final working concentrations of 50 μg/ml, 20 μg/mL, 5 μg/mL, and 1 μg/mL were freshly prepared from the stock in AdDMEM just before incubation with sporozoites.

### Invasion and replication inhibition assays

MDBK cells were seeded in four different 96-well plates (one per each harvested time point) at a density of 0.05×10 ^6^ cells per well and incubated for four hours at 37 ^o^C, 5% CO _2_. Right after, sporozoites (0.2×10 ^6^ per well) of *E. tenella* Wis strain were pre-incubated for 1 h at 41 ^o^C, 5% CO _2_ with each anticoccidial compound concentration; untreated sporozoites were used as controls for invasion and replication. After pre-incubation, sporozoites were centrifuged at 1,500 g for 10 minutes at room temperature and washed with PBS to rinse them free of drugs, then resuspended in 300 μl of AdDMEM (equivalent to 100 μl per well). Sporozoites were then added to MDBK monolayers seeded in 96-well plates (after the 4 hours incubation period) and incubated at 41 ^o^C, 5% CO _2_. All the wells were washed twice in AdDMEM to remover extracellular sporozoites. The first time point was harvested at two hours post infection (hpi) by washing twice with PBS, then adding RTL buffer (Qiagen) and storing the plate at -20 ^o^C until used for genomic DNA isolation. The other infected monolayers were collected and stored in the same way at 24, 44 and 52 hpi. Three wells were used per condition (for untreated and treated sporozoites and for each drug concentration at each time point). Two whole separated experiments carried out in different weeks were performed.

### Isolation of nucleic acids

Genomic DNA (gDNA) was isolated from the samples stored in RTL buffer (Qiagen) using the AllPrep DNA/RNA 96 Kit (Qiagen) following manufacturer’s instructions using the vacuum manifold for processing 96-well plates (Qiagen).

### Real-time quantitative PCR (qPCR)

CFX96 Touch R Real-Time PCR Detection System (Bio-Rad) was used to perform the quantitative PCR following the procedures described previously, using DNA-binding dye SsoFastTM EvaGreen Supermix (Bio-Rad) ( Marugan-Hernandez *et al.*, 2016). For parasite quantification, the number of haploid genomes (equivalent to single sporozoites or merozoites) per well (three wells/sample, technical replicates) was determined for each condition using gDNA specific primers for *E. tenella* 5S rDNA (Fw_5S: TCATCACCCAAAGGGATT, Rv_5S: TTCATACTGCGTCTAATGCAC) ( Clark *et al.*, 2008) and a standard curve of sporozoite gDNA extracted by the same methods (gDNA equivalent to 10 ^7^ followed by serial dilution until 10 ^2^). Once the run was completed, a baseline was calculated by Bio-Rad CFX Manager software (Bio-Rad) and applied to each sample to compare the quantification cycles (Cq values) obtained from different wells.

### Data analysis

The number of sporozoites for each data point analysed by qPCR was automatically determined for each well by a regression analysis using Bio-Rad CFX Manager software (Bio-Rad). Three replicates were included per sample to allow single points showing a standard deviation of more than 0.5 to be excluded from the analysis without affecting the quantification, if all three curves were out of this range, qPCR was repeated for this sample.

*In vitro* inhibition percentage for each anticoccidial drug and concentration was calculated following the equation implemented by Thabet *et al.* (2017):

$$\text{\% of Inhibition}=100\;\times \;\left(1-\frac{\text{number of}\;\text{E. tenella}\;\text{gene copies in treated sample}}{\text{number of}\;\text{E. tenella}\;\text{gene copies in non}-\text{treated control}}\right)$$

Comparison of replication rates after drug treatment were done by calculating slopes of the regression line ( *m*) between 24 and 44 hpi, which were calculated following the universal formula:

$$m\;\left(\text{slope}\right)=\frac{y\left(\text{44 hpi}\right)-y\left(\text{24 hpi}\right)}{20\;\left(\text{time lag}\right)}$$

## Results

### Sporozoites of *E. tenella* invaded and replicated at equivalent rates when transferred to a 96-well plate format

**Figure 1.**
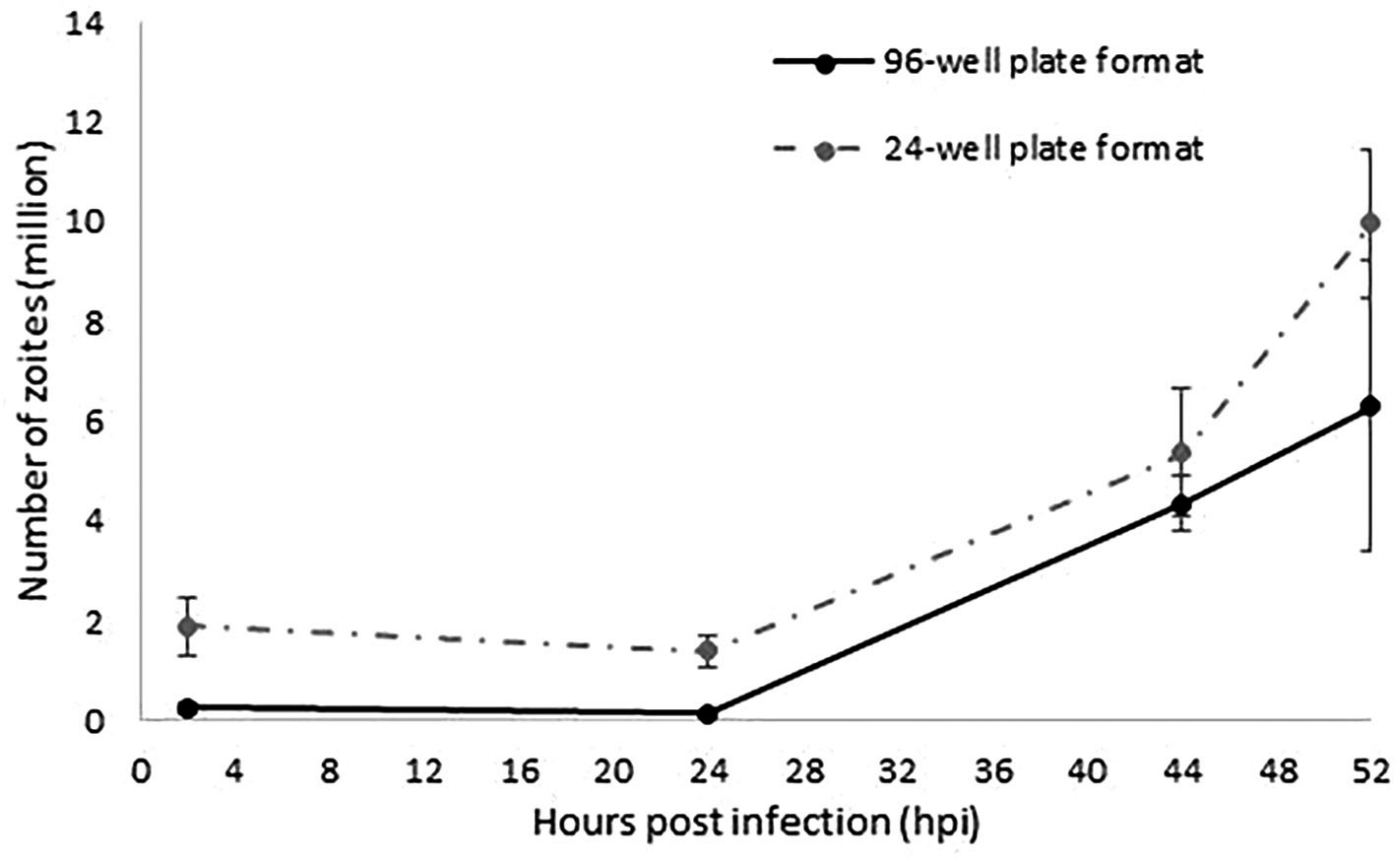
Evaluation of *E. tenella* invasion and endogenous development by qPCR. The continuous line represents the number of zoites quantified by qPCR in samples collected at 2, 24, 44 and 52 hpi as the average of the two different replicated experiments performed in separated weeks in a 96-well plate format. Discontinuous line represents data extracted from Marugan-Hernandez *et al.* (2020) equivalent to the number of zoites quantified by qPCR at the same time points as the average of the three different replicated experiments performed in separated weeks in a 24-well plate format. Error bars represent the standard deviation between repeated experiments for each plate format. Both types of plate format show an equivalent linear fashion growth after 24 hpi.

For the miniaturisation of the model to a 96-well plate format, we screened out the optimal combination of MDBK cells and *E. tenella* sporozoites. Good monolayer confluences were achieved with 0.05×10 ^6^ cells/well. An optimal rate of invasion and development without causing multiple sporozoite infection per cell was obtained with a 4:1 sporozoites:cell ratio (0.2×10 ^6^ sporozoites/well). Parasite invasion and development was evaluated in a time course experiment followed by qPCR ( Figure 1). Increasing amounts of parasite DNA were detected from 24 hpi onwards as described for the 24-well format, indicative of nuclear replication. The linear rate of replication was equivalent to the one described in a 24-well plate format ( Figure 1), validating in this way the suitability of a 96-well plate format to track and evaluate sporozoites invasion and replication. Higher variability was found for the latest time point (52 hpi) which is explained by the rupture of schizonts which will release merozoites as has been observed before ( Marugan-Hernandez *et al*., 2020); these merozoites are washed from the monolayers and therefore not contributing to the parasite numbers when analysed by qPCR. Fewer parasite number were detected for the 96-well plate format along the time course, which correlated with the lower initial numbers of sporozoites used for monolayers infection (0.2 million/well versus 1 million/well).

### *Eimeria tenella* 96-well plate format *in vitro* model is suitable to evaluate inhibition of invasion and development after treatment with traditional anticoccidials

Pre-treatment of sporozoites with different concentrations of AMP, ROB and SAL, three drugs with reported anticoccidial activity, showed different degrees of inhibition of invasion and replication ( Table 3). The pre-treatment with AMP at any concentration caused a moderate inhibition of invasion at 2 and 24 hpi (10-45%; average of 22%); however, those sporozoites which were successful at invasion then developed at the same rate as the untreated controls (slope of the regression curve was parallel to that exhibited by the untreated control). Pre-treatment with ROB had little effects on invasion (inhibition of 0-20%, average 5.6%); nevertheless, sporozoites did not replicate well once intracellularly (slope decreased compared with untreated controls, with sporozoite numbers decreasing for the highest concentration, 50 μg/mL). Pre-treatment with SAL exhibited effects in both invasion and development. Invasion was inhibited at higher levels than for AMP (10-60%; average 27%) and intracellular replication was severely impaired for the higher concentrations (50 and 20 μg/mL). In general, for every drug, higher inhibition of invasion and replication was observed for the higher concentration, although some variations were observed. These results evidence the suitability of the miniaturised model to detect inhibition of invasion and/or replication.

**Table 3.** Percentages of inhibition of invasion (2-24 hpi) and replication (44-52 hpi) and replication rate (slope between 24-44 hpi) of *E. tenella* sporozoites after pre-treatment with anticoccidial dugs amprolium hydrochloride (AMP), robenidine hydrochloride (ROB) and salinomycin monosodium hydrate (SAL) at different concentrations (50, 20, 5, 1 μg/ml). Each value represents the average of the percentage of inhibition±standard deviation or slope of two different experiments including each three wells per condition, calculated using the equations displayed in *Data analysis* section.

Average	Concentration (μg/ml)	2 hpi	24 hpi	44 hpi	52 hpi	Slope (24-44 hpi) *
**AMP**	**50**	22.05±21.71	34.22±34.22	8.17±8.17	47.03±15.18	282486
	**20**	17.34±10.96	7.17±7.17	12.88±12.88	23.54±23.54	264669
	**5**	22.89±4.85	10.19±10.19	0.00±0.00	14.11±6.08	227343
	**1**	46.78±5.45	24.45±24.45	1.96±1.96	0.00±0.00	227636
**ROB**	**50**	9.13±9.13	25.71±25.71	5.13±5.13	6.05±6.05	-9013
	**20**	0.00±0.00	7.91±7.91	97.81±0.07	48.49±48.49	100536
	**5**	0.00±0.00	9.07±9.07	45.43±23.7	40.99±31.24	194893
	**1**	0.00±0.00	0.00±0.00	5.42±3.62	0.00±0.00	89050
**SAL**	**50**	62.42±1.79	19.02±19.02	52.34±13.91	18.23±18.23	-3380
	**20**	16.47±16.47	7.94±7.94	98.4±0.4	99.23±0.09	4664
	**5**	40.02±6.5	13.5±13.5	94.19±0.04	87.87±0.94	55507
	**1**	44.18±21.42	31.35±14.43	73.66±6.83	49.41±20.62	152050

White squares – no inhibition; Pale grey squares – low degree of inhibition; Grey squares – mild degree of inhibition; Dark grey squares - strong degree of inhibition.

* Slope average value for untreated sporozoites: 309,189.

## Discussion

In this study, the *in vitro* model developed to evaluate compounds with potential anticoccidial effects on sporozoites of *E. tenella* has been successfully miniaturised from a 24-well plate format to a 96-well format. Conditions were refined to create an optimal confluence of the monolayer supporting invasion rates of >70% in the new format. These conditions have proved to support schizonts and merozoite development as described before in the original model ( Marugan-Hernandez *et al.*, 2020). The tracking of the sporozoites invasion and development by qPCR in time course experiments have shown the same linear growth fashion than described before and the evaluation of traditional anticoccidial drugs to validate the model has also proved the suitability of this model to detect different levels of inhibition of invasion and/or development.

*Eimeria* parasites are self-limited monoxenous (single-host) parasites, and despite many efforts, there are no efficient *in vitro* systems supporting continuous replication or the completion of the life cycle; in consequence, research on this species depends on the use of animals. Examination of the literature shows that testing of novel anticoccidial compounds, mainly natural products, is in expansion. In 54 refereed publications between 2017 and 2021 a total of 19,786 chickens (~4,000 per annum) were used to evaluate novel compounds for anticoccidial effects. In addition to these chickens already mentioned, there are chickens used in unpublished studies from pharmaceutical/nutrition companies or those only providing negative results, numbers of which are harder to discover. Looking specifically at some 2021 publications, if pre-screening with *in vitro* models had been used by Fu *et al.* (2021), concentrations could have been adjusted to reduce animals from 630 to 480 (23.8%). Similarly, the use of *in vitro* models could have excluded one testing group for both Lima *et al.* (2021) and Herrero-Encinas *et al.* (2021), reducing animals from 900 to 720 (20%) and 400 to 320 (20%), respectively. If we extrapolate these data (an average of 20% reduction) to the total published studies in 2021, the use of our *in vitro* models could have avoided the experimental use of 792 chickens (out of 3,958) globally, or 3,958 (out of 19,786) in the last 5 years. Nevertheless, we still need some chickens to generate enough parasite materials to perform the *in vitro* pre-screenings. Therefore, the described modification to the *in vitro* model will have a direct impact on the reduction of animal use.

Testing one compound at four different concentrations (excluding untreated controls) requires 12 million of sporozoites in the 24-well plate format whereas only 2.4 million are necessary in the 96-well plate one. Efficiency of sporozoite excystation and purification from oocysts is set at 1:1 in our experimental settings. A single chicken can produce an average of 30 million oocysts without causing clinical symptoms. Therefore, with the use of one chicken, we could test at least ten compounds (plus untreated compounds) with the 96-well format, whereas only two compounds (plus controls) could be tested in the 24-well plate format. This supposes a direct 80% reduction in animal use for the evaluation of anticoccidial compounds locally. However, since we have collaborations with several research institutions and companies at different locations worldwide, this miniaturised model will also have a significant global impact on the number of chickens undergoing a mild severity procedure for the generation of *E. tenella* oocysts.

Another advantage of this miniaturisation is the high-throughput capacity to test many compounds simultaneously, while reducing materials, reagents and time to perform them. Up to seven compounds could be tested on a single plate per time point (including controls) using <10 mL of culture media, versus only two compounds per plate using volumes of >20 mL per plate in the 24-well plate format. Time is also significantly reduced by a streamlined protocol using 96-well plates and multichannel pipettes throughout the whole experiment, from cell culture to DNA isolation and qPCR analysis. These added benefits will also impact positively in the wider uptake of the model by other researchers since costs and time to achieved results will be significantly reduced.

## Data availability

### Underlying data

DRYAD: Reduction of chickens use to perform in vitro pre-screening of novel anticoccidials by miniaturisation and increased throughput of the current *Eimeria tenella* compound-screening model, https://doi.org/10.5061/dryad.mw6m90605 ( Marugan-Hernandez & Arias, 2022).

This project contains the following files:

- Experiment_1.xlsx
- Experiment_2.xlsx
- 1st_EXPERIMENT_-_2hpi_DMSO_-_ROB_20.pcrd
- 1st_EXPERIMENT_-_2hpi_ROB_5_-_24hpi_AMP_20.pcrd
- 1st_EXPERIMENT_-_24hpi_AMP_5_-_SAL_20.pcrd
- 1st_EXPERIMENT_-_24hpi_SAL_5_-_44hpi_ROB_20.pcrd
- 1st_EXPERIMENT_-_44hpi_ROB_5_-_52hpi_AMP_20.pcrd
- 1st_EXPERIMENT_-_52hpi_AMP_5_-_SAL_20.pcrd
- 1st_EXPERIMENT_-_52hpi_SAL_5-1___Controls.pcrd
- EXP_2._2hpi_DMSO-ROB_20.pcrd
- EXP_2._2hpi_ROB_5_-_24hpi_DMEM.pcrd
- EXP_2._24hpi_AMP_50_-_ROB_1.pcrd
- EXP_2._24hpi_SAL_50_-_44hpi_AMP_20.pcrd
- EXP_2._44hpi_AMP_5_-_SAL_20.pcrd
- EXP_2._44hpi_SAL_5_-_52hpi_AMP_1.pcrd
- EXP_2._52hpi_ROB_50_-_SAL_1.pcrd
- README.txt

Data are available under the terms of the Creative Commons Zero “No rights reserved” data waiver (CC0 1.0 Public domain dedication).
