# Supplementary material for: Reduction of chickens use to perform in vitro pre-screening of novel anticoccidials by miniaturisation and increased throughput of the current Eimeria tenella compound-screening model
Source: F1000Res. 2024 Oct 1;11:1135. Originally published 2022 Oct 4. [Version 2] doi: 10.12688/f1000research.123102.2 (PMC11806246; doi:10.12688/f1000research.123102.2)
Supplement: Peer review history

# Reviewer report: Reviewer response for version 2

**DOI:** 10.5256/f1000research.172468.r356737

**Published:** 2025-02-07

**Authors:** Benjamin Adjei-Mensah

**Affiliations:** 1 Department of Animal Science, University of Ghana School of Agriculture (Ringgold ID: 356390), Accra, Greater Accra Region, Ghana

I would like to appreciate the author for addressing all the comments I suggested. The article report is in good shape for indexing. Thanks.

Are the 3Rs implications of the work described accurately?

Yes

Is the work clearly and accurately presented and does it cite the current literature?

Yes

If applicable, is the statistical analysis and its interpretation appropriate?

Yes

Are all the source data underlying the results available to ensure full reproducibility?

Yes

Is the study design appropriate and is the work technically sound?

Yes

Are the conclusions drawn adequately supported by the results?

Yes

Are a suitable application and appropriate end-users identified?

Yes

Are sufficient details of methods and analysis provided to allow replication by others?

Yes

Reviewer Expertise:

Nutrition, Gut physiology, Poultry science

I confirm that I have read this submission and believe that I have an appropriate level of expertise to confirm that it is of an acceptable scientific standard.

# Reviewer report: Reviewer response for version 2

**DOI:** 10.5256/f1000research.172468.r340270

**Published:** 2024-12-23

**Authors:** Xiaokai Song

**Affiliations:** 1 Nanjing Agricultural University, Nanjing, China

In this manuscript, the author provides a detailed description of the miniaturization of the experimental model for the invasion of *Eimeria tenella* sporozoite in MDBK cells. The miniaturized model was proven by in vitro experiments to allow for simultaneous pre-screening of a greater number of different concentrations of anti-coccidial drugs, which will further reduce the number of infected chickens required for these drugs to be tested experimentally. Overall, this study presents a valuable contribution to the field of anticoccidial drug research. The authors’ efforts to reduce animal usage and improve the efficiency of drug screening are commendable.

 I have the following comments:

1. Conduct a more comprehensive statistical analysis, including: Comparison of drug efficacy between the 24-well and 96-well plate formats at each time point and overall inhibition rate. Analysis of the reproducibility of the 96-well plate model across multiple experiments. Expand the discussion to address the limitations of the 96-well plate model compared to the 24-well plate model. Provide more details on the logistics and potential challenges of the pooled resource approach for oocyst amplification. Consider including a section on the potential for adapting the 96-well plate model for other Eimeria species.
2. The title of Table 3 is too lengthy; it is suggested to be more concise.

Is the work clearly and accurately presented and does it cite the current literature?

Yes

If applicable, is the statistical analysis and its interpretation appropriate?

Yes

Are all the source data underlying the results available to ensure full reproducibility?

Yes

Is the study design appropriate and is the work technically sound?

Yes

Are the conclusions drawn adequately supported by the results?

Yes

Are sufficient details of methods and analysis provided to allow replication by others?

Yes

Reviewer Expertise:

The Mechanism of Invasion by Chicken Coccidia

I confirm that I have read this submission and believe that I have an appropriate level of expertise to confirm that it is of an acceptable scientific standard.

# Reviewer report: Reviewer response for version 1

**DOI:** 10.5256/f1000research.135172.r173894

**Published:** 2023-11-13

**Authors:** Joseph D. Turner

**Affiliations:** 1 Centre for Drugs and Diagnostics, Department of Parasitology, Liverpool School of Tropical Medicine, Liverpool, UK

The work of Arias-Maroto *et al.,* details miniaturisation of an *Eimeria tenella *sporozoite invasion and replication assay in immortalised bovine kidney epithelial cells. The *in vitro *assay, if successful in emulating the larger plate system, would further cut down the number of experimentally infected chickens required to generate parasites for these drug assays.

3Rs statement

 When stating the 80% reduction metric of use of chickens for generation of parasites due to miniaturisation, it would be informative if the authors could estimate numbers of chickens used for this purpose per annum in their lab, in the UK or globally that may be reduced via adoption of a miniaturised assay, as they have done in the discussion.

Methods

- Rationale for selection of seeding densities in 96-well plates needs more explanation - was it based on scaling of volume, well area or well volume compared with 24 well plate, for instance?
- Readers may wish to understand why it is necessary to culture at 41 degrees centigrade for infection of MDBK with sporozoites as this is unusual for mammalian cell culture.
- A flow chart of the invasion and replication inhibition assays would help clarify timings of when sporozoites and drugs were added / removed, as without careful reading this is hard to follow.

Results and conclusions

 I agree the data supports the conclusion that infection and growth rate of *Eimeria *is similar between optimised 24 and 96 well plate cultures, but a suitable statistical comparison would be needed to confirm this.

 Further, there is a lack of statistics to test whether any of the drug compounds significantly inhibited invasion and/or growth - using PCR quantifications at either time point or the overall growth rate. 

 Further, there is no discussion whether these drugs and concentrations tested induce similar or different levels of inhibition in the miniaturised 96-well system compared with the prior standardised 24 well plate system.

 Without statistical tests of the 3 replicates per condition to demonstrate meaningful levels of drug efficacy, and how reproducible between different experiments, and how consistent cf the 24-well original screen, it is not currently possible to conclude the 96-well plate system 'has been successfully miniaturised'.  

Suggest revisions before considering the work fully valid:

1. Please test that *Eimeria *infectivity / growth is not significantly different in 96- v 24-well plate systems.
2. Please could the authors provide statistical tests to determine which drugs significantly reduce invasion and/or replication in their miniaturised assay.
3. Could the authors compare drug efficacy achieved in 24 vs 96 well assays with any or all drug compounds screened?

Are the 3Rs implications of the work described accurately?

Partly

Is the work clearly and accurately presented and does it cite the current literature?

Yes

If applicable, is the statistical analysis and its interpretation appropriate?

No

Are all the source data underlying the results available to ensure full reproducibility?

Yes

Is the study design appropriate and is the work technically sound?

Partly

Are the conclusions drawn adequately supported by the results?

Partly

Are a suitable application and appropriate end-users identified?

Yes

Are sufficient details of methods and analysis provided to allow replication by others?

Partly

Reviewer Expertise:

Parasitology, drug discovery & development, infection biology, immunobiology. Referee suggested by the NC3Rs for their scientific expertise and experience in assessing 3Rs impact.

I confirm that I have read this submission and believe that I have an appropriate level of expertise to state that I do not consider it to be of an acceptable scientific standard, for reasons outlined above.

# Response

**Published:** 2024-09-24

**Authors:** Virginia Marugan-Hernandez

**Affiliations:** Royal Veterinary College, London, UK

We would like to thank Joseph D. Turner for his time and effort reviewing our manuscript. We provide below responses for the different queries and issues raised.

- **Rationale for selection of seeding densities in 96-well plates needs more explanation - was it based on scaling of volume, well area or well volume compared with 24 well plate, for instance?**

The two densities were derived of the previously optimised number for the 24-well plate format that was conferring a 100% confluence without an excess of cells (Marugan-Hernandez et al., 2020). This has been now detailed in ‘Optimisation of infection ratios’ section:

 ‘ *Based on the amount of 0.3×10 ^6^ per well in 500* *μ* *l, previously optimised for the 24-well plates format to confer a confluence of 100% without excess of cells (where the surface of each well is four time larger than in the 96-well plate format; 0.3×10 ^6^ / 4 = 0.075×10 ^6^), we tested 0.1×10 ^6^ and 0.05×10 ^6 ^cells per well for the 96-well plate format (just above and below to the equivalent figure)*.’

- **Readers may wish to understand why it is necessary to culture at 41 degrees centigrade for infection of MDBK with sporozoites as this is unusual for mammalian cell culture.**

The temperature in the chicken intestine is 41 ^o^C and this is the optimal temperature for *Eimeria tenella* development, if the temperature is lower, the parasite will not replicate. This has been clarified in ‘Optimisation of infection ratios’ section:

 ‘ *This temperature is necessary for parasite development, as chicken Eimeria parasites develop in the intestine of chickens, whose internal temperature is 41 ^o^C, higher than mammalian hosts. MDBK cells can support this increased temperature for up to six/seven days, longer than the required time for in vitro parasite replication.*’

- **A flow chart of the invasion and replication inhibition assays would help clarify timings of when sporozoites and drugs were added / removed, as without careful reading this is hard to follow.**

A flow chart has been included (Figure 1) aimed to clarify the experimental design of the invasion and replication assays.

*‘Figure 1. Flow chart of sporozoite i* *nvasion and replication inhibition assays.*

***1.*** *MDBK cells were seeded into 96-well plates to form a confluent monolayer (0.05x10 ^6^ cells/well); **2.** Sporozoites were purified from oocysts using DE-52 columns (Pastor-Fernandez et al., 2019); **3.** Sporozoites were pre-incubated with anticoccidial drugs at four different concentrations (or without drugs for controls) for 1 hour at 4 ^o^C; **4.** Sporozoites were centrifuged, resuspended in AdDMEM and added to MDBK monolayers to allow invasion. **5.** Infected cell monolayers were lysed and collected; **6.** Genomic DNA was extracted from collected infected cell monolayers; **7.** Real time qPCR was used to evaluate the number of parasite genomes for each sample.’*

- **I agree the data supports the conclusion that infection and growth rate of Eimeria is similar between optimised 24 and 96 well plate cultures, but a suitable statistical comparison would be needed to confirm this.**

A statistical comparison using the non-parametric Spearman's rank test has been included. This has shown a high degree of correlation with r=1. All this has been detailed in ‘Data analysis’ and ‘Sporozoites of *E. tenella* invaded and replicated at equivalent rates when transferred to a 96-well plate format’ sections:

 ‘ *Correlation (r) between sporozoite quantification between the 24 and 96-well plate format cultures was determined using the nonparametric Spearman's rank test using* *GraphPad Prism version 6.00 (GraphPad Software, La Jolla, California, USA)*.’

 ‘ *The linear rate of replication showed a very strong correlation between the 96 and the 24-well plate format models (r=1; 95% confidence interval;* *Figure* *2)*’

- **Further, there is a lack of statistics to test whether any of the drug compounds significantly inhibited invasion and/or growth - using PCR quantifications at either time point or the overall growth rate.**

Statistical analysis have been included to evaluate differences in the ‘number of zoites’ between the different groups. This has now been detailed in ‘Data analysis’ and ‘ *Eimeria tenella* 96-well plate format *in vitro* model is suitable to evaluate inhibition of invasion and development after treatment with traditional anticoccidials’ sections. A new figures (Figure 3) has been added.

*‘Comparison of groups evaluating differences in ‘number of zoites’ after anticoccidial drug treatment vs. controls was performed using non-parametric Kruskal-Wallis test. The analysis was followed by Dunnett’s multiple comparisons as a post-hoc test. Statistically significant differences were established using a p<0.05. All analyses were performed using GraphPad Prism version 6.00.’*

*‘Pre-treatment of sporozoites with different concentrations of AMP, ROB and SAL, three drugs with reported anticoccidial activity, showed a relative dose dependant effect ( Figure 3). There were no significant differences in the number of sporozoites able to invade host cells after the pre-treated groups vs. the controls at either 2 and 24 hpi ( Figure 3, A and B). However, treatment with the highest dose of AMP (50* *μ* *g/ml) show a significant reduction in replication at both 44 and 52 hpi. Similarly, treatment with ROB (50* *μ* *g/ml) exhibited a significant reduction in parasite numbers at 52 hpi ( Figure 3, C and D).’*

*‘* *Figure 3. Effects of anticoccidial drugs in intracellular numbers of parasites.*

*A and B. Graphs shows the number of parasites by qPCR after invasion at 2 and 24 hpi, respectively (before nuclear division has started, see Figure 2); C and D. Graphs shows the number of parasites by qPCR after nuclear replication at 44 and 52 hpi, respectively. X-axis numbers represent the specific drug dilution used in* *μ* *g/ml. Asterisks indicate significant differences with the DMEM/DMSO controls (Dunnett’s multiple comparison test p<0.05).’*

- **Further, there is no discussion whether these drugs and concentrations tested induce similar or different levels of inhibition in the miniaturised 96-well system compared with the prior standardised 24 well plate system.**

We have included the comparison of anticoccidial drugs pre-treatment between the 24 and 96-well plate format in the ‘Discussion’ section:

*‘When the same anticoccidial drugs (AMP, ROB and SAL) were evaluated in the 24-well plate format, only concentrations of 5* *μ* *g/ml were tested ( Marugan-Hernandez et al., 2020). Comparing this same concentration in the 96-well plate format, AMP and SAL showed equivalent effects in parasite numbers in invasion and replication for both models. AMP did not show any significant change regarding the controls at any point, whereas SAL did not show differences in invasion, but replication was significantly reduced at 44 hpi (Two-way ANOVA, followed by Dunnett’s post-hoc test). ROB at 5* *μ* *g/ml did not correlate with previous results from the 24-well plate model where strong effects were found in both invasion and replication. In the 96-well plate format, ROB did not show any effect in invasion or replication at 5* *μ* *g/ml. Interestingly, this concentration has shown inhibitory effects in subsequent studies ( Felici et al., 2023) performed in the 96-well plate format, that were equivalent to the 24-well plate format described before. We attributed the lack of significant effects from ROB at 5* *μ* *g/ml in the current study to a loss of effect due to continue use of the drug (where stock has gone several freeze-thaw rounds), which we have observed after using it as inhibition control in further studies.’*

  

- **Without statistical tests of the 3 replicates per condition to demonstrate meaningful levels of drug efficacy, and how reproducible between different experiments, and how consistent cf the 24-well original screen, it is not currently possible to conclude the 96-well plate system 'has been successfully miniaturised'. **
- **Please test that Eimeria infectivity / growth is not significantly different in 96- v 24-well plate systems.**
- **Please could the authors provide statistical tests to determine which drugs significantly reduce invasion and/or replication in their miniaturised assay.**
- **Could the authors compare drug efficacy achieved in 24 vs 96 well assays with any or all drug compounds screened?**

All these points have been now included in the new version of the manuscript. Detailed responses can be seen above.

# Reviewer report: Reviewer response for version 1

**DOI:** 10.5256/f1000research.135172.r210464

**Published:** 2023-11-02

**Authors:** Benjamin Adjei-Mensah

**Affiliations:** 1 Department of Animal Science, University of Ghana School of Agriculture (Ringgold ID: 356390), Accra, Greater Accra Region, Ghana

Brief report title: ***Reduction of chickens use to perform in vitro pre-screening of novel anticoccidials by miniaturisation and increased throughput of the current Eimeria tenella compound screening***

 This brief report aimed to adhere to the 3R, focusing much on the reduction aspect by reducing the number of chickens involved in the study. The goal was to use a high number of well plates (96) to pre-screen 10 compounds using a single chicken against a previous study of 24 well plates, which pre-screened two compounds per chicken.

 The article is clear and cites relevant literature to back the claims of the findings of this study. Appropriate experimental design has been followed and therefore makes the work relevant in the academic space. The statistical approach and its interpretation are appropriate for readability and comprehension. Based on the data provided and the findings, this work can be reproduced. Additionally, the conclusions drawn are supported by the findings of the study.

 However, I found these minor issues that need to be attended to:

1. The article still requires English Language editing (proofreading).
2. The authors should be clear on the number of replications. Were all the 10 birds induced with the oocysts? If so, was it the only one that was used during the in vitro studies?
3. Check that there is space between all the reported values and their SI units.
4. I expected some comparisons to be made in the discussion of the findings of the synthetic compounds against that which was reported using the essential oils since the former already have public health issues as indicated in the introduction.
5. The authors should be clear on the number of replications. Were all the 10 birds induced with the oocysts? If so, was it the only one that was used during the in vitro studies?
6. Check that there is space between all the reported values and their SI units.
7. I expected some comparisons to be made in the discussion of the findings of the synthetic compounds against that which was reported using the essential oils since the former already have public health issues as indicated in the introduction.

Are the 3Rs implications of the work described accurately?

Yes

Is the work clearly and accurately presented and does it cite the current literature?

Yes

If applicable, is the statistical analysis and its interpretation appropriate?

Yes

Are all the source data underlying the results available to ensure full reproducibility?

Yes

Is the study design appropriate and is the work technically sound?

Yes

Are the conclusions drawn adequately supported by the results?

Yes

Are a suitable application and appropriate end-users identified?

Yes

Are sufficient details of methods and analysis provided to allow replication by others?

Yes

Reviewer Expertise:

Nutrition, Gut physiology, Poultry science

I confirm that I have read this submission and believe that I have an appropriate level of expertise to confirm that it is of an acceptable scientific standard, however I have significant reservations, as outlined above.

# Response

**Published:** 2024-09-24

**Authors:** Virginia Marugan-Hernandez

**Affiliations:** Royal Veterinary College, London, UK

We would like to thank Benjamin Adjei-Mensah for his time and effort reviewing our manuscript. We provide below responses for the different queries and issues raised.

**Reviewer comment: **The article still requires English Language editing (proofreading).

**Author response: **The article has gone further proofreading, changes have been highlighted throughout the manuscript using ‘Track Changes’ tool.

**Reviewer comment: **The authors should be clear on the number of replications. Were all the 10 birds induced with the oocysts? If so, was it the only one that was used during the in vitro studies?

**Author response: **We would like to clarify that the chickens are used to generate oocysts that would be later used to release the sporozoite stage to proceed with the *in vitro* infections. There are no ‘replicates’ in these type of experiments, as there is uniformity between the different batches of productions as we use a reference strain (not field isolates). For oocyst amplification, groups of 10 animals are typically used, and to minimize the overall numbers, the research group operates a pooled resource so that each batch is utilized efficiently, with minimal wastage (this means that when a batch has been produced, this will be used by different researchers for different experiments, and when is needed, a new round of amplification will be done). Chickens will be orally infected with 4,000 oocyst/animal, with an expected yield of ~300 - 500 x 10 ^6^ sporulated oocysts per 10 chickens. All this details are contained in Table 1. In order to clarify this, some text has been added in ‘Parasites and chickens’ section:

 ‘ *Eimeria tenella* *Wisconsin (Wis) strain (* *McDougald & Jeffers, 1976* *) was propagated in ten four-week-old White Leghorn chickens reared under specific pathogen-free conditions. Each chicken was inoculated with 4,000 oocysts to obtain sufficient oocysts for all the in vitro experimental procedures.* *Table 1* *summarises the detailed information of the experiment and used animals following ARRIVE guidelines.*’

 Based on this yields, we did the estimation that the use of one chicken (which would produce ~30 x 10 ^6^ oocysts) would allow the evaluation of 10 compounds with a 96-well plate format versus; whereas this will only allow to evaluate two compounds with a 24-well plate format, as the number of sporozoites (and therefor oocysts) needed per well is much higher. More details for this has been also added on the ‘Discussion’ section:

 ‘ *Testing one compound at four different concentrations (excluding untreated controls) requires 12 million of sporozoites in the 24-well plate format (4 concentrations x 3 wells x 1 million/well) whereas only 2.4 million are necessary in the 96-well plate one (4 concentrations x 3 wells x 0.2 million/well); this means the miniaturised model requires 5 times less parasite material. Efficiency of sporozoite excystation and purification from oocysts is set at 1:1 in our experimental settings. A single chicken can produce an average of 30 million oocysts without causing clinical symptoms. Therefore, with the use of one chicken, we could test at least ten compounds (2.4 million/compound x 10 = 24 million oocysts, plus untreated compounds) with the 96-well format, whereas only two compounds (12 million/compound x 2 = 24 million oocysts, plus controls) could be tested in the 24-well plate format. This supposes a direct 80% reduction in animal use (testing 10 compounds would require 5 chickens in the 24-well plate format versus only 1 in the 96-well plate format, which means 4 less chickens out of 5), for the evaluation of anticoccidial compounds locally*.’

 Regarding the in vitro experiments. The number of replicates are specified in ‘Invasion and replication inhibition assays’ section:

 ‘ *Two different biological replicated were performed in different weeks*.’

**​​​​​​​Reviewer comment: **Check that there is space between all the reported values and their SI units.

**Author response: **This has been done (highlighted by the ‘Track Changes’ tool).

**Reviewer comment:** I expected some comparisons to be made in the discussion of the findings of the synthetic compounds against that which was reported using the essential oils since the former already have public health issues as indicated in the introduction.

**Author response: **Previous studies using the 24-well plate format to evaluate essential oils from oregano, garlic, thyme and sage (Sidiropoulou et al., 2020-2022) only focused in invasion. In this studies, high dosed of essential oils (100 μg/ml) show similar effects to Robenidine at 5 μg/ml

 However, as we did no evaluate any essential oil in our current study, we did not include any of these findings on the discussion. As we have recently published the evaluation of essential oils in the 96-well plate format, a small paragraph has been included in this regard in the ‘Discussion’ section:

*‘This 96-well plate model has already supported the evaluation of essential oils and their main active compounds (* *Felici et al., 2023* *), where the formers have shown similar effects on invasion and replication than ROB and SAL. This is important as research in natural compounds it is essential to replace current anticoccidials for which several resistances have been developed in field strains threatening their efficacy.* *These latest results evidence the suitability of the miniaturised model to detect inhibition of invasion and/or replication of natural compounds in addition to classical anticoccidal drugs.’*
